# Association of α-klotho with subclinical carotid atherosclerosis in subjects with type 1 diabetes mellitus

**DOI:** 10.1186/s12933-022-01640-3

**Published:** 2022-10-11

**Authors:** Esmeralda Castelblanco, Marta Hernández, Nuria Alonso, Aina Ribes-Betriu, Jordi Real, Minerva Granado-Casas, Joana Rossell, Marina Idalia Rojo-López, Adriana Silvia Dusso, Josep Julve, Didac Mauricio

**Affiliations:** 1 Endocrinology, Metabolism and Lipid Research Division, Department of Medicine, Washington University School of Medicine, 63110 St Louis, MO USA; 2 Unitat de Suport a la Recerca Barcelona, Institut Universitari d’Investigació en Atenció Primària Jordi Gol i Gurina (IDIAP Jordi Gol), 08007 Barcelona, Spain; 3 Department of Endocrinology & Nutrition, Hospital Universitari Arnau de Vilanova & Institut d’Investigació Biomédica de Lleida (IRB Lleida), 25198 Lleida, Spain; 4 CIBER de Diabetes y Enfermedades Metabólicas Asociadas, Instituto de Salud Carlos III, 28029 Madrid, Spain; 5 Department of Endocrinology & Nutrition, Hospital Universitari Germans Trias i Pujol, 08916 Badalona, Spain; 6 Department of Medicine, Autonomous University of Barcelona, 08916 Barcelona, Spain; 7 Institut d’Investigació Biomèdica Sant Pau (IIB Sant Pau), 08041 Barcelona, Spain; 8 Department of Endocrinology & Nutrition, Hospital de la Santa Creu i Sant Pau, 08041 Barcelona, Spain; 9 Faculty of Medicine, University of Vic (UVIC/UCC), 08500 Vic, Spain

**Keywords:** Type 1 diabetes mellitus, Mineral metabolism, Fibroblast growth factor 23, α-klotho

## Abstract

**Background:**

Compelling evidence suggests that the fibroblast growth factor 23 (FGF23) / α-klotho axis is impaired in subjects with diabetes mellitus. We examined the relationship between parameters related to calcium/phosphate homeostasis, including FGF23 and α-klotho, and subclinical carotid atherosclerosis burden in type 1 diabetes mellitus (T1D) subjects.

**Methods:**

This cross-sectional study involved 226 subjects with T1D and 147 age-, sex- and plaque-matched, non-diabetic (non-T1D) subjects, both with normal renal function. Carotid ultrasound was performed to determine the presence and burden of atheromatous plaques. Concentrations of the intact form of FGF23 and α-klotho were assessed by ELISA. Calcium, phosphate, parathyroid hormone, and vitamin D levels were also determined. Negative binomial regression models were used to examine relationship between parameters studied and subclinical carotid atherosclerosis.

**Results:**

Only FGF23 was increased in T1D compared with non-diabetic subjects (> 2-fold; *p* < 0.05). α-klotho was higher in subjects with subclinical carotid atherosclerosis (1.4-fold, *p* < 0.05). Regression analysis revealed that the log α-klotho concentration was positively associated with the presence of subclinical carotid atherosclerosis both in T1D subjects (incidence rate ratio [IRR]: 1.41; 95% confidence interval [CI], 1.06–1.89; *p* < 0.05) and in non-T1D subjects (IRR: 1.65; 95% CI, 1.02–2.75; *p* < 0.05). The models also showed that age, smoking and albuminuria-to-creatinine ratio were positively associated with subclinical carotid atherosclerosis in T1D subjects. Interestingly, sex-related protection against plaque was also revealed in T1D women.

**Conclusion:**

Higher α-klotho was associated with subclinical carotid atherosclerotic in the absence of kidney dysfunction. This finding also points to a new pathophysiological pathway involved in the development and progression of this complication.

**Supplementary Information:**

The online version contains supplementary material available at 10.1186/s12933-022-01640-3.

## Background

Mineral metabolism disturbances and impaired kidney function are frequent features in diabetic subjects [1]. Adverse cardiovascular outcomes and altered mineral metabolism in subjects with impaired insulin signaling have been attributed to kidney dysfunction [2–4].

The Fibroblast growth factor 23 (FGF23)/α-klotho axis is heavily impaired in chronic kidney disease (CKD) [5]. Low concentrations of α-klotho and high concentrations of FGF23 generally predict the degree of kidney dysfunction at early CKD stages, cardiovascular disease, and death in both subjects with CKD and the general population [5]. Despite the generally accepted inverse relationship between circulating α-klotho and vascular disease in CKD patients [6], it has been refuted by some recent reports [7, 8].

Decreased concentrations of serum α-klotho have also been associated with increased carotid artery intima-media thickness (cIMT), a surrogate of subclinical carotid atherosclerosis [9], in healthy subjects without impaired kidney function [10]. In this context, α-klotho remained independently associated with metabolic syndrome, a condition commonly associated with impaired insulin signaling and cardiovascular disease [11], even after adjusting for age, sex, estimated glomerular filtration rate (eGFR), and overt proteinuria [12]. In addition, FGF23 elevations have been recently associated with increased cardiovascular risk in the absence of apparent clinical kidney disease [13–15].

The relationship between altered mineral metabolism components, including FGF23/α-klotho, and atherosclerosis in subjects with type 1 diabetes mellitus (T1D) without previous cardiovascular events and kidney impairment is supported by different lines of evidence. First, decreased vitamin D levels in subjects with T1D has been associated with worse cardiovascular outcomes [16] and coronary artery calcification [17]. Second, circulating vitamin D and FGF23 levels showed, respectively, negative, and positive associations with arterial stiffness in T1D subjects [18]. Third, elevations in parathyroid hormone (PTH), which together with FGF23 keep serum concentrations of phosphate under tight control [19, 20], have also been related to the development of cardiovascular disease [21]. Furthermore, PTH concentrations have been positively associated with arterial stiffness in T1D subjects, even after adjustment for additional risk factors, including eGFR [22]. Finally, recent studies have shown that lower levels of α-klotho in subjects with T1D are associated with increased values of cIMT in the absence of apparent signs of renal dysfunction as revealed by normal values of creatinine clearance [23].

Our research group has recently found that the calcium phosphate product was related to subclinical carotid atherosclerosis in subjects with type 2 diabetes mellitus without apparent kidney dysfunction [28]. Moreover, alterations in the concentrations of both FGF23 and α-klotho have been frequently related to disturbances in mineral metabolism due to an impaired kidney function [5]. However, analysis of the relationship of both FGF23 and α-klotho with atherosclerotic cardiovascular disease has been lacking in the majority, if not all, reports [24–27]. Indeed, the relationship, if any, of the main mineral metabolism factors (i.e., calcium, phosphate, 25-hydroxyvitamin D (vitamin D), FGF23, a-klotho, PTH) with subclinical atherosclerosis burden in T1D subjects, in the absence of kidney impairment has not been assessed yet. Thus, the intended purpose of this sub-study was to uncover novel relationships between mineral metabolism-related biomarkers (calcium, phosphate, PTH, vitamin D, FGF23, and α-klotho) and subclinical carotid atherosclerosis in T1D subjects who were free of renal impairment and previous cardiovascular events.

## Methods

### Study design and participants’ selection

This study was a cross-sectional, post-hoc analysis of a previous study designed to assess subclinical carotid atherosclerosis in subjects with T1D [24]. Selected participants included 226 subjects with T1D, and 147 age-, sex- and plaque-matched, non-diabetic subjects with complete data of calcium, phosphate, PTH, and vitamin D levels, and a plasma sample available to assess FGF23 and α-klotho concentrations.

The inclusion criteria were age > 18 years old, with T1D duration of at least 1 year, with no evidence of previous cardiovascular disease, defined as any form of clinical coronary heart disease, stroke, or peripheral vascular disease (this included the diagnosis of diabetic foot disease), and normal renal function (eGFR > 60 mL/min/1.73 m^2^; albuminuria-to-creatinine ratio (ACR) < 30 mg/g). Subjects with established CKD (defined as eGFR < 60 mL/min/1.73 m^2^; albuminuria-to-creatinine ratio (ACR) ≥30 mg/g), prior treatment with vitamin D and/or calcium supplements, and previous clinical signs of cardiovascular disease were excluded. For the non-T1D group, inclusion and exclusion criteria were the same except for the T1D diagnostic variables, thus, these subjects had fasting glucose and HbA1c values below 100 mg/dL and 5.7%, respectively. Clinical records from subjects were thoroughly reviewed in addition to an anamnestic evaluation and physical examination. The study was conducted in accordance with the tenets of the Declaration of Helsinki, and approval was obtained from the ethics committees of both participating centers. Written informed consent was provided by all participants.

### Clinical and laboratory procedures

Demographic and clinical data were obtained from each participant. Weight, height, and waist circumference were measured using standard clinical procedures. Subjects were classified as having hypertension or dyslipidemia when they were receiving antihypertensive or lipid-lowering treatment, respectively. Biochemical variables were determined in fasting blood and urine samples. All biochemical analyses were performed using standard laboratory methods. Basic blood and urine biochemistry were determined using commercial kits adapted to a Hitachi Modular DDPP analyzer (Roche Diagnostics, Indianapolis, USA). HbA1c was measured using an HPLC Variant II Turbo (Bio-RAD, Hercules, USA). Intact PTH was measured in an Elecsys E170 analyzer (Roche Diagnostics, Indianapolis, USA) by electrochemiluminescence immunoassay. The eGFR was calculated using the Chronic Kidney Disease Epidemiology Collaboration equation [25]. Plasma concentrations of the intact form of FGF23 (TECOmedical AG, Sissach, Switzerland) and α-klotho (Cusabio Biotech Co. Ltd., Wuhan, China) were measured by an enzyme-linked immunosorbent assay. Serum vitamin D concentrations were measured using an Architect i2000SR analyzer (Abbott Diagnostics, Lake Forest, USA) by a chemiluminescent microparticle immunoassay. Vitamin D deficiency was defined as a concentration below 20 ng/mL, and vitamin D insufficiency as values between 20 and < 30 ng/mL, in accordance with the Endocrine Society guidelines [26]. Dietary intake of vitamin D and calcium was evaluated using a 101-food-item semiquantitative food frequency validated questionnaire (available at http://bibliodieta.umh.es/files/2011/07/CFA101.pdf) [27].

### Carotid ultrasonography imaging

Subclinical atherosclerosis was identified by non-invasive image analysis (carotid ultrasound) as the presence and number of atherosclerotic plaques in the carotid arteries. All carotid ultrasound protocols were performed by the same researcher who was blinded to the characteristics of the study subjects. Ultrasonography imaging of the common and internal carotid arteries was performed using a Siemens Sequoia 512 and a 15 MHz linear array probe. The presence and number of plaques was identified using 2D-mode and color Doppler imaging, and these techniques were similarly used in longitudinal and transverse planes. A detailed description of the ultrasound procedures used in this study has been recently described [24, 28].

### Statistical analysis

Quantitative variables were presented as mean and standard deviation, median and interquartile intervals or number (percentage), unless otherwise indicated. Qualitative variables were summarized using absolute or relative frequencies. Bivariate analysis consisting of the chi-squared or McNemar tests were used to compare qualitative variables and the *t*-test or Mann–Whitney *U* test were used to compare quantitative variables using compareGroups R package [29]. The association between α-klotho (in subjects with T1D and controls stratified) and subclinical carotid atherosclerosis (expressed as number of atherosclerotic plaques detected) was assessed using fitted negative binomial regression models. Negative binomial regression is similar to regular multiple regression, except for the fact that the dependent (Y) variable is an observed count. Thus, the possible values of Y are the non-negative integers: 0, 1, 2, 3, and so on (in this analysis, the number of atherosclerotic plaques) [30]. Negative binomial regression is a generalization of Poisson’s regression that loosens the restrictive assumption that the variance is equal to the mean made by the Poisson model; thus, we used this approach to achieve more robust estimations [31]. The models were performed using the *glm.nb* function from MASS R Package [32]. Clinically relevant variables, as potential confusion covariables, further α-klotho, were included into 3 models, as a confirmatory analysis. The association measures were estimated and were expressed by incidence rate ratios (IRR) and their 95% confidence interval (95% CI). The statistical analysis was carried out using R 4.1.0 [33] (https://www.R-project.org/). A *p*-value less than 0.05 was considered statistically significant.

## Results

Demographic, clinical, and basic biochemical data of subjects with and without T1D are shown in Table 1. Because the participants in this sub-study were matched for age, sex, and atherosclerotic plaque, these variables did not differ between both groups of subjects. The number of atherosclerotic plaques did not differ between groups.

Adiposity surrogates (i.e., BMI and waist circumference) did not significantly differ among both groups. Systolic blood pressure, but not diastolic blood pressure, was significantly elevated in the T1D group (127 mm Hg) compared with non-diabetic subjects (121 mm Hg); accordingly, the percentage of subjects with hypertension was significantly increased in the T1D group (24.3%) compared with the non-T1D group (6.1%).

The percentage of subjects with T1D treated for dyslipidemia was higher (38.1%) than that observed in non-T1D subjects (10.2%) (Table 1). In line with taking lipid-lowering medications, the serum concentrations of total triglycerides, total cholesterol and LDL cholesterol were significantly reduced in the subjects with T1D.

The eGFR did not differ between groups (Table 1), with filtrate values over 60. Likewise, the ACR was similar in both groups.

**Table 1:** Demographic and biochemical characteristics and atherosclerosis-related parameters of the subjects included in the study

Variables	Non-T1D	T1D	***p***-value	N
***Clinical data***	**N = 147**	** N = 226**		
Age	43.8 (10.9)	44.6 (10.8)	0.514	373
Sex (women)	84 (57.1%)	120 (53.1%)	0.509	373
Diabetes duration (years)		21.4 (10.7)		226
Ethnicity (Caucasian)	145 (98.6%)	224 (99.1%)	0.648	373
Tobacco exposure			0.773	371
Former smoker	37 (25.5%)	53 (23.5%)		
Smoker	41 (28.3%)	60 (26.5%)		
Hypertension	9 (6.1%)	55 (24.3%)	< 0.001	373
Dyslipidemia	15 (10.2%)	86 (38.1%)	< 0.001	373
Systolic blood pressure (mm Hg)	121 (14.2)	127 (18.1)	< 0.001	366
Diastolic blood pressure (mm Hg)	74.4 (9.6)	74.7 (10.0)	0.771	366
BMI (kg/m^2^)	25.0 (4.0)	25.8 (4.0)	0.071	373
Waist circumference (cm)	88.6 (12.0)	89.0 (12.3)	0.796	357
Diabetic Retinopathy			< 0.001	373
Healthy	147 (100%)	0 (0%)		
Diabetic Retinopathy		89 (39.4%)		
No Diabetic Retinopathy		137 (60.6%)		
*Biochemistry*				
HbA1c (%)	5.34 (0.31)	7.64 (1.01)	< 0.001	372
Triglycerides (mg/dL)	95.8 (56.8)	75.7 (37.5)	< 0.001	373
Total cholesterol (mg/dL)	195 (37)	183 (32)	< 0.001	373
LDL cholesterol (mg/dL)	116 (32)	103 (27)	< 0.001	371
HDL cholesterol (mg/dL)	61.8 (18.9)	64.2 (16.1)	0.200	372
Creatinine (mg/dL)	0.78 (0.15)	0.77 (0.15)	0.589	373
ACR (mg/g)	6.00 (9.63)	8.24 (15.2)	0.083	373
eGFR (mL/min/1.73 m^2^)	104 (14)	103 (13)	0.908	373
Microalbuminuria	8 (5.52%)	12 (5.80%)	1.000	352
*Atherosclerosis imaging*				
Atherosclerotic Plaque			0.902	
One plaque	16 (11%)	28 (12%)		
Multiple plaques	16 (11%)	25 (11%)		

Data are presented as mean (standard deviation), or n (%), as appropriate. ACR, albuminuria-to-creatinine ratio; BMI, body mass index; eGFR, estimate glomerular filtration rate based on the CKD-EPI (Chronic Kidney Disease Epidemiology Collaboration) equation; HDL, high-density lipoprotein; LDL, low-density lipoprotein

Among the different mineral metabolism components analyzed, serum calcium, but not phosphate, was significantly decreased in subjects with T1D compared with non-T1D subjects (Table 2). Of note, the levels of FGF23 were significantly elevated (∼2-fold, *p* < 0.05) in subjects with T1D. Neither the levels of PTH or α-klotho, nor vitamin D differed between groups.

**Table 2:** Mineral metabolism parameters in non-diabetic and T1D subjects

	Non-T1D	T1D	***p***-value	N
	N = 147	N = 226		
Calcium (mg/dL)	9.41 (0.35)	9.28 (0.38)	0.001	373
Phosphate (mg/dL)	3.56 (0.60)	3.54 (0.63)	0.733	373
Vitamin D (ng/mL)	21.6 (8.2)	21.0 (11.4)	0.570	373
PTH (pmol/L)	4.84 (1.70)	4.69 (1.67)	0.396	373
FGF23 (pg/mL)	34.9 (19.3)	72.8 (167.0)	0.001	373
α-klotho (ng/mL)	0.19 (0.21)	0.20 (0.27)	0.515	373

Data were presented as mean (standard deviation). FGF23, fibroblastic growth factor 23; PTH, parathyroid hormone; Vitamin D, 25-hydroxyvitamin D

The sub-analysis by sex revealed that α-klotho was significantly increased in women compared with men irrespective of whether they were in the T1D or non-T1D group. In contrast, FGF23 did not differ between sexes (Supplementary Table 1).

The subjects with subclinical carotid atherosclerosis were older (age mean: +10.3 years old) and more obese (waist circumference mean: +5.6 cm) than those without plaque(s) (Table 3). Although the values of eGFR and ACR were worse in subjects with plaques, they fell into the clinically normal range. Mean systolic blood pressure was higher (+ 9 mm Hg) in subjects with plaques than in those without. In line with this, a higher proportion of subjects with plaques were receiving medication for hypertension than those without plaque. Finally, total cholesterol was elevated (*p* = 0.04) in the subjects with atherosclerotic plaques, with this being mainly explained by a concomitant, though marginal (*p* = 0.076), elevation in LDL cholesterol. More subjects with plaque were medicated for dyslipidemia than those without plaque (52.9% vs. 19.4%, *p* < 0.001).

**Table 3:** Subanalysis of demographic and biochemical characteristics, including mineral metabolism-related parameters, in subjects with and without subclinical carotid atherosclerosis

	No plaque	Plaque	***p***-value	N
***Clinical data***	N = 288	N = 85		
Age	41.9 (10.3)	52.2 (8.8)	< 0.001	373
Sex (women)	159 (55.2%)	45 (52.9%)	0.806	373
T1D number, (%)	173 (60.1)	53 (62.4)	0.801	373
Ethnicity (Caucasian)	285 (99.0%)	84 (98.8%)	1.000	373
Tobacco exposure			0.278	371
Former smoker	69 (24.0%)	21 (25.0%)		
Smoker	73 (25.4%)	28 (33.3%)		
Hypertension	28 (9.7%)	36 (42.4%)	< 0.001	373
Dyslipidemia	56 (19.4%)	45 (52.9%)	< 0.001	373
Systolic blood pressure (mm Hg)	123 (15.7)	132 (19.1)	< 0.001	366
Diastolic blood pressure (mm Hg)	74.4 (9.7)	75.4 (10.2)	0.434	366
BMI (kg/m^2^)	25.1 (3.9)	26.9 (3.9)	< 0.001	373
Waist circumference (cm)	87.6 (11.9)	93.2 (12.0)	< 0.001	357
Diabetic Retinopathy			0.004	373
Healthy controls	115 (39.9%)	32 (37.6%)		
Diabetic Retinopathy	58 (20.1%)	31 (36.5%)		
No Diabetic Retinopathy	115 (39.9%)	22 (25.9)		
*Biochemistry*				
HbA1c (%)	6.67 (1.39)	6.97 (1.35)	0.072	372
Triglycerides (mg/dL)	81.2 (45.6)	92.0 (51.1)	0.081	373
Total cholesterol (mg/dL)	185 (32)	195 (39)	0.040	373
LDL cholesterol (mg/dL)	107 (27)	114 (35)	0.076	371
HDL cholesterol (mg/dL)	63 (18)	63 (16)	0.912	372
Creatinine (mg/dL)	0.77 (0.15)	0.78 (0.15)	0.442	373
Albuminuria-to-creatinine ratio (mg/g)	6.2 (10.8)	11.1 (19.3)	0.027	373
eGFR (mL/min/1.73 m^2^)	106 (13)	96 (12)	< 0.001	312
Microalbuminuria	13 (4.78%)	7 (8.75%)	0.178	352
*Mineral metabolism*				
FGF23 (pg/mL)	56.7 (124.0)	62.0 (157.0)	0.777	373
α-klotho (ng/mL)	0.18 (0.25)	0.26 (0.24)	0.010	373
Calcium (mg/dL)	9.33 (0.35)	9.34 (0.43)	0.941	373
Phosphate (mg/dL)	3.55 (0.60)	3.53 (0.68)	0.854	373
Vitamin D (ng/mL)	21.2 (8.7)	21.3 (14.3)	0.971	373
PTH (pmol/L)	4.73 (1.67)	4.82 (1.72)	0.679	373

Data were presented as mean (standard deviation), or n (%). BMI, body mass index; eGFR, estimate glomerular filtration rate based on the CKD-EPI (Chronic Kidney Disease Epidemiology Collaboration) equation. FGF23, fibroblastic growth factor 23; HDL, high-density lipoprotein; LDL, low-density lipoprotein; PTH, parathyroid hormone; Vitamin D, 25-hydroxyvitamin D

Notably, the concentrations of α-klotho were significantly higher (1.4-fold, *p* = 0.01) in subjects with subclinical carotid atherosclerosis than in those without (Table 3). No further differences in any other component of mineral metabolism were observed.

**Table 4:** Binomial negative regression models to identify factors associated with the subclinical carotid atherosclerosis burden in subjects with T1D.

	Model 1		Model 2		Model 3	
**Predictors**	**IRR**	**95% CI**	**IRR**	**95% CI**	**IRR**	**95% CI**
α-klotho (log scale)	1.40 *	1.06–1.86	1.41 *	1.06–1.89	1.41 *	1.06–1.89
Sex (women)	0.57	0.33–0.97	0.54 *	0.31–0.95	0.55 *	0.31–0.97
Age	**1.08 ‡**	1.05–1.11	**1.08 ‡**	1.05–1.11	**1.08 ‡**	1.05–1.11
sBP	1.00	0.98–1.01	1.00	0.99–1.01	1.00	0.99–1.01
BMI	1.04	0.98–1.11	1.04	0.98–1.11	1.04	0.98–1.11
Smoker	**2.39 †**	1.37–4.22	**2.28 †**	1.29–4.07	**2.29 †**	1.28–4.13
Former smoker	0.96	0.49–1.82	0.98	0.51–1.85	1.00	0.52–1.88
Hypertension	1.71	0.98–3.02	1.65	0.93–2.93	1.65	0.93–2.92
Dyslipidemia	1.53	0.93–2.55	1.62	0.95–2.80	1.60	0.94–2.77
ACR	**1.01 ‡**	1.00-1.02	**1.02 †**	1.01–1.03	**1.02 †**	1.00-1.03
Calcium			0.93	0.47–1.88	0.95	0.47–1.94
Phosphate			1.15	0.73–1.83	1.13	0.71–1.81
PTH			0.94	0.80–1.10	0.93	0.79–1.10
FGF23					1.00	1.00–1.00
Vitamin D					1.00	0.98–1.01
Observations	225		225		225	
R^2^ Nagelkerke	0.574		0.578		0.580	

Association measure expressed by **Incidence rate ratio** (IRR) and 95% confidence interval (95% CI). ACR, Albuminuria-to-creatinine ratio; BMI, body mass index; FGF23, fibroblastic growth factor 23; PTH, parathyroid hormone; sBP, systolic blood pressure; Vitamin D, 25-hydroxyvitamin D

* *p* < 0.05; † *p* < 0.01; ‡ *p* < 0.001

α-klotho was positively associated with subclinical atherosclerotic disease (IRR: 1.41; 95% CI, 1.06–1.89) (Table 4), even after additional adjustment for other potential confounders (model 3). α-klotho also showed a positive association with subclinical atherosclerotic disease (IRR: 1.65; 95% CI, 1.02–2.75; *p* < 0.05) in a subanalysis only considering non-diabetic participants (Supplementary Table 2).

Multivariable linear regression analysis also identified other independent factors associated with plaque in subjects with T1D (Table 4). Despite the doubling of FGF23 levels in subjects with T1D (Table 2), this phosphaturic hormone was not associated with an increased risk of plaque (Table 4). The models also showed that age, tobacco exposure and ACR were associated with the number of atherosclerotic plaques in T1D subjects (Table 4). However, only age and ACR were associated with the number of atherosclerotic plaques in non-T1D subjects (Supplementary Table 2). Notably, ACR remained significantly associated with plaque even in subjects displaying clinically normal kidney function (Table 4, Supplementary Table 2).

Another variable positively associated with subclinical carotid atherosclerosis was medication for hypertension and dyslipidemia though only in non-T1D subjects (Table 4, Supplementary Table 2).

## Discussion

The potential of mineral metabolic disturbances, including FGF23, α-klotho, PTH, phosphate, and vitamin D status, as non-traditional biomarkers of atherosclerosis is still under debate. Since kidney disease positively influences mineral metabolism and hence cardiovascular risk [34], we specifically analyzed the relationship between different mineral metabolism-related biomarkers for subclinical carotid atherosclerosis in T1D subjects with physiologically normal values of eGFR and without previous cardiovascular events. The results revealed that α-klotho was associated with subclinical carotid atherosclerosis burden in T1D subjects. Interestingly, α-klotho concentrations were also associated with the number of atherosclerotic plaques in subjects without T1D. Our data strikingly differed from those from other studies reporting that lower, rather than higher, concentrations of serum α-klotho are related to increased atherosclerotic cardiovascular risk [35–38].

Serum α-klotho concentrations are positively influenced by different intrinsic factors. Consistent with other reports [36, 39, 40], our data showed concomitant elevations of α-klotho in females. Although the rationale for such sex-specific differences is poorly defined, they could be related to hormonal differences. However, according to our data, sex (women) was not negatively associated with the presence and number of atherosclerotic plaques in T1D subjects. In line with this, the sex ratio did not significantly differ between the group of subjects with plaque compared with those without plaque, and thus this parameter did not help to explain the observed positive relationship between plasma α-klotho and the presence of subclinical carotid atherosclerosis.

We want to underline that FGF23 is increased in subjects with obesity [41]. In our study, both BMI and waist circumference were significantly increased in subjects with atherosclerotic plaques (Table 3). However, BMI was not associated with carotid atherosclerosis in subjects with T1D (Table 4) or in non-diabetic subjects. (Supplementary Table 2). Unfortunately, we did not have complete data from all study subjects regarding waist circumference. Nevertheless, as waist is better measure of abdominal obesity [42], we decided to explore this potential association. Therefore, using the subset of subjects with available data, we performed an additional analysis including waist instead of BMI in the model (data not shown). This analysis did not reveal any difference from the model that included BMI as a measure of adiposity. However, we strongly believe adiposity, especially abdominal obesity measures, should be included in future research on this matter.

Serum α-klotho concentrations are also positively influenced by extrinsic factors. Statins and angiotensin II blockade are positive regulators of the gene expression α-klotho [43, 44]. Whether circulating α-klotho may be indirectly augmented by antihypertensive or lipid-lowering agents needs to be explored in future studies. Notably, the relative proportion of subjects with subclinical carotid atherosclerosis taking lipid-lowering agents and angiotensin II receptor blockers was significantly increased compared with those without plaque (anti-dyslipidemic medication: 52.9% vs. 19.4%; antihypertensive medication: 42.4% vs. 9.7%) (Table 3), and hence they were overrepresented in the group of subjects with plaque. Thus, our data might be suggesting that α-klotho concentrations could be increased in response to lipid-lowering or antihypertensive treatments and may indirectly reflect adherence to medications. However, the binomial negative regression analysis revealed that medication for either dyslipidemia or hypertension was only marginally associated with subclinical carotid atherosclerosis burden in T1D subjects (Table 4), reaching incidence rate values close to those calculated for α-klotho. Intriguingly, the calculated associations between lipid-lowering or antihypertensive treatments and plaque were exacerbated in non-T1D subjects (Model 3, Supplementary Table 2). To directly check the potential interaction between the lipid-lowering or antihypertensive medications and α-klotho concentrations and its relationship with the number of atherosclerotic plaques, we further repeated the binomial negative regression analysis but this time after adjusting for the intake of such treatments in T1D subjects (Supplementary Table 3). Our analysis showed that the positive association of α-klotho with subclinical carotid atherosclerosis remained significant in this subanalysis, suggesting the participation of other mechanisms still not identified to explain such a relationship.

Some strengths and limitations of our study deserve additional comments. Among the strengths, our study is the first considering simultaneously evaluation of multiple factors associated with calcium/phosphate homeostasis, including FGF23, α-klotho, PTH, vitamin D, phosphate, and calcium in subjects with T1D with a thorough clinical characterization. Further, some limitations should also be acknowledged. First, this study’s observational and cross-sectional nature does not allow us to ensure complete control of all the potential (still unknown) confounding factors, especially those related to the increase of α-klotho in participants with subclinical carotid atherosclerosis. Moreover, the study design also fails to examine cause-effect relationships or the temporary association between α-klotho and atherosclerotic plaque burden, but allows an estimation of the likelihood of having plaque in those subjects with elevated α-klotho. Second, the lack of urinary phosphorous levels impeded the assessment of a critical renal response to the FGF23/α-klotho axis responsible for phosphorous retention and its consequent inflammatory component. High circulating α-klotho without phosphaturia would indicate α-klotho dysfunction as occurs with its proatherogenic impact in these subjects. Third, urinary and circulating concentrations of parameters of mineral metabolism are profoundly affected by the menopausal status. Unfortunately, the menopausal status was not available from women of this cohort. Finally, we acknowledge that both FGF23 and α-klotho are not currently used in clinical practice; however, we believe that further research is needed to gain further insight in the translational and clinical significance of the relationship between α-klotho and subclinical atherosclerosis in T1D, and the potential application of this protein as a biomarker.

## Conclusion

Our data suggest that higher α-klotho concentration could be a marker of subclinical carotid atherosclerosis burden, pointing to a new pathophysiological pathway involved in the development and progression of this complication in T1D subjects. Additional population studies on mineral metabolism-related biomarkers are warranted to validate the association and to establish the mechanism involved between these parameters and subclinical carotid atherosclerosis in this population.

## Electronic supplementary material

Below is the link to the electronic supplementary material.

Supplementary Material 1

## Data Availability

Proposals relating to the data access should be directed to the corresponding authors. To gain access, data requestors will need to sign a data access agreement.

## Abbreviations

ACR: Albuminuria-to-creatinine ratio
BMI: body mass index
cIMT: carotid artery intima-media thickness
CKD: chronic kidney disease
dBP: diastolic blood pressure
eGFR: estimate glomerular filtration rate
FGF23: fibroblastic growth factor 23
HDL: high density lipoprotein
LDL: low density lipoprotein
PTH: parathyroid hormone
sBP: systolic blood pressure
Vitamin D: 25-hydroxyvitamin D
