# Supplementary material for: Association of α-klotho with subclinical carotid atherosclerosis in subjects with type 1 diabetes mellitus
Source: Cardiovasc Diabetol. 2022 Oct 11;21:207. doi: 10.1186/s12933-022-01640-3 (PMC9554979; doi:10.1186/s12933-022-01640-3)
Supplement: Supplementary file 1 — Supplementary Material 1 [file 12933_2022_1640_MOESM1_ESM.docx]

**Supplementary Table 1.** Subanalysis of FGF23 and α-klotho in men and women.

|  | **Men** | **Women** | ***p*-value** |
| --- | --- | --- | --- |
| *T1D subjects (N=226)* | N = 106 | N = 120 |  |
| FGF23 (pg/mL) | 75.7 (188) | 70.3 (148) | 0.809 |
| α-klotho (ng/mL) | 0.17 (0.17) | 0.27 (0.32) | 0.001 |
| *Non-T1D subjects (N=147)* | N = 63 | N = 84 |  |
| FGF23 (pg/mL) | 37.9 (22.7) | 32.7 (16.1) | 0.129 |
| α-klotho (ng/mL) | 0.14 (0.15) | 0.25 (0.22) | 0.001 |

Data are presented as mean (standard deviation). FGF23, fibroblastic growth factor 23.

**Supplementary Table 2.** Binomial negative regression models to identify factors predicting the subclinical carotid atherosclerosis burden in non-T1D subjects.

|  | **Model 1** | | **Model 2** | | **Model 3** | |
| --- | --- | --- | --- | --- | --- | --- |
| **Predictors** | **IRR** | **95% CI** | **IRR** | **95% CI** | **IRR** | **95% CI** |
| α-klotho (log scale) | **1.53 ^a^** | 0.94-2.58 | **1.53 ^b^** | 0.94-2.56 | **1.65 *** | 1.02-2.75 |
| Sex (women) | 0.51 | 0.19-1.30 | 0.49 | 0.18-1.24 | 0.43 | 0.16-1.10 |
| Age | **1.06 †** | 1.01-1.11 | **1.06 *** | 1.01-1.13 | **1.05 ^c^** | 1.00-1.11 |
| sBP | 0.99 | 0.96-1.03 | 0.99 | 0.96-1.03 | 0.98 | 0.95-1.02 |
| BMI | 1.09 | 0.98-1.22 | 1.08 | 0.97-1.21 | 1.05 | 0.95-1.18 |
| ACR | 1.02 | 0.97-1.06 | 1.01 | 0.97-1.06 | 1.01 | 0.97-1.05 |
| Smoker | 1.92 | 0.72-5.23 | 1.81 | 0.67-5.01 | 1.21 | 0.45-3.26 |
| Former smoker | 1.98 | 0.67-6.08 | 1.87 | 0.62-5.82 | 1.74 | 0.60-5.19 |
| Hypertension | 2.23 | 0.68-7.91 | 2.24 | 0.68-7.91 | **5.88 †** | 1.57-24.31 |
| Dyslipidemia | **3.10 *** | 1.09-9.11 | **3.45*** | 1.20-10.36 | **3.82 *** | 1.41-10.69 |
| Calcium |  |  | 0.53 | 0.18-1.51 | **0.30 *** | 0.09-0.91 |
| Phosphate |  |  | 0.71 | 0.38-1.30 | **0.52 *** | 0.27-0.99 |
| PTH |  |  | 0.91 | 0.69-1.17 | 0.80 | 0.60-1.05 |
| FGF23 |  |  |  |  | 0.98 | 0.96-1.01 |
| Vitamin D |  |  |  |  | **0.90 *** | 0.83-0.96 |
| Observations | 141 |  | 141 |  | 141 |  |
| R^2^ Nagelkerke | 0.431 |  | 0.455 |  | 0.553 |  |

Association measure expressed by **Incidence rate ratio** (IRR) and 95% confidence interval (95% CI). ACR, Albuminuria-to-creatinine ratio; BMI, body mass index; Dyslipidemia, treatment for dyslipidemia; FGF23, fibroblastic growth factor 23; hypertension; treatment for hypertension; PTH, parathyroid hormone; Vitamin D, 25(OH)D, 25-hydroxyvitamin D.

* *p*<0.05; † *p*<0.01;

^a^ *p*=0.071; ^b^ *p*=0.076: ^c^ *p*=0.069

**Supplementary Table 3.** Binomial negative regression model showing the predictive value of α-klotho on the subclinical carotid atherosclerosis burden in T1D subjects adjusting for anti-hypertensive and lipid-lowering medication.

| **Predictors** | **IRR** | **95% CI** |
| --- | --- | --- |
| **α-klotho (log scale)** | **1.58 *** | **1.01-2.49** |
| **Sex (women)** | **0.56 ^a^** | **0.32-1.00** |
| **Age** | **1.08 ‡** | **1.05-1.11** |
| sBP | 1.00 | 0.99-1.01 |
| BMI | 1.04 | 0.97-1.11 |
| ACR | 1.02 | 1.00-1.03 |
| **Smoker** | **2.26 †** | **1.26-4.08** |
| Former smoker | 0.97 | 0.50-1.85 |
| Hypertension | 1.47 | 0.52-4.19 |
| Dyslipidemia | 1.25 | 0.43-3.75 |
| Calcium | 0.97 | 0.48-1.98 |
| Phosphate | 1.12 | 0.70-1.80 |
| PTH | 0.93 | 0.79-1.09 |
| FGF23 | 1.00 | 1.00-1.00 |
| Vitamin D | 1.00 | 0.98-1.01 |
| α-klotho (log scale):dyslipidemia | 0.87 | 0.49-1.53 |
| α-klotho (log scale):hypertension | 0.94 | 0.55-1.59 |
| Observations | 225 |  |
| R^2^ Nagelkerke | 0.582 |  |

Association measure expressed by **Incidence rate ratio** (IRR) and 95% confidence interval (95% CI). ACR, Albuminuria-to-creatinine ratio; BMI, body mass index; DLP, treatment for dyslipidemia; FGF23, fibroblastic growth factor 23; PTH, parathyroid hormone; Vitamin D, 25(OH)D, 25-hydroxyvitamin D.

* *p*<0.05; † *p*<0.01; ‡ *p*<0.001

^a^ *p*=0.05
